# Comparison of Anxiety and Depression Symptoms in Individuals According to their Sex, Type of Cardiac Device, and Diagnosis of Chagas Disease

**DOI:** 10.21470/1678-9741-2021-0392

**Published:** 2022

**Authors:** Carina Aparecida Marosti Dessotte, Elisa Maia de Oliveira Grotti, Isabelle Brigliadori Ignácio, Paolla Algarte Fernandes, Suellen Rodrigues de Oliveira Maier, Lidia Aparecida Rossi, Rosana Aparecida Spadoti Dantas

**Affiliations:** 1 Department of General and Specialized Nursing, Escola de Enfermagem de Ribeirão Preto, Universidade de São Paulo, Ribeirão Preto, São Paulo, Brazil.; 2 Escola de Enfermagem de Ribeirão Preto, Universidade de São Paulo, Ribeirão Preto, São Paulo, Brazil.; 3 Faculdade de Ciências da Saúde, Universidade Federal de Rondonópolis, Rondonópolis, Mato Grosso, Brazil.

**Keywords:** Defibrillators, Chagas Disease, Anxiety, Depression, Equipment and Supplies, Weights and Measures, Surveys and Questionnaires

## Abstract

**Introduction:**

Implantable cardiac pacemakers or cardioverter defibrillators are alternatives for the treatment of arrhythmias, however, their use has caused changes in the emotional state of patients. The objective of this study was to compare the measures of anxiety and depression symptoms in individuals according to their sex, type of cardiac device, and diagnosis of Chagas disease.

**Methods:**

This is an observational and cross-sectional study conducted with adults with implantable cardiac pacemakers or cardioverter defibrillators. Data was collected using a sociodemographic and clinical questionnaire and the Hospital Anxiety and Depression Scale. We used the Student’s t-test for independent samples and the Chi-squared test, with a significance level of 0.05.

**Results:**

Two hundred forty-four patients participated in the study, 168 with cardiac pacemakers and 76 with implantable cardioverter defibrillators; 104 had Chagas cardiomyopathy (85 with cardiac pacemakers and 19 with implantable cardioverter defibrillators). No statistically significant differences were found in measures of anxiety and depression symptoms according to device type (P=0.594 and P=0.071, respectively) and the presence of Chagas etiology (P=0.649 and P=0.354, respectively). Women had higher mean scores for anxiety (P=0.002) and depression symptoms (P<0.001).

**Conclusion:**

In the comparison between the groups, according to the type of implanted device and the diagnosis of Chagas disease, no significant differences were found in the measures of anxiety and depression symptoms. Women showed higher means when compared to men, indicating the need to test and implement interventions to minimize these symptoms in this population.

**Table:** 

Abbreviations, Acronyms & Symbols	
AVB	= Atrioventricular block
HADS	= Hospital Anxiety and Depression Scale
ICD	= Implantable cardioverter defibrillator
PM	= Pacemaker
SD	= Standard deviation

## INTRODUCTION

In Brazil, it is estimated that more than 20 million people have some type of cardiac arrhythmia. In 2019, of 5,740 hospitalizations for conduction disorders and cardiac arrhythmias, 2,740 occurred in the southeast region^[1]^. Tissue damage to the heart or changes in the electrical signals that control the heartbeat, caused by disease, trauma, or genetic alterations, can lead to changes in heart rhythm.

Chagas disease is one of the conditions that can result in changes in the gastrointestinal and cardiac systems^[2]^. Chronic Chagas cardiomyopathy, an inflammatory and progressive immune response, can lead to myocardial fibrosis, heart failure, atrial and sinus node dysfunction, and intraventricular block, causing symptomatic or non-symptomatic arrhythmias^[2]^. In the chronic phase of this disease, chronic fibrosis myocarditis compromises myocardial contractility and the conduction system of the heart, leading to alterations in heart rhythm^[2]^.

Chagas myocarditis is the most common form of cardiomyopathy in Latin American countries, consisting of a serious public health problem for endemic areas. Although the incidence of Chagas disease is declining in Brazil, even in rural areas and among the low-income population, this disease is a public health concern, since it entails serious problems because of its long-term consequences for individuals and society and due to the fact that, with immigration, it has spread to other countries^[3]^.

Chagas disease is still one of the conditions that lead to the use of cardiac pacemakers (PMs) and defibrillators in Brazil, exceeding the indications due to coronary artery disease in some regions^[4]^. However, its use has psychological implications and discomfort because it requires living with a biomechanical device permanently attached to the body. Besides the feelings of threat to life increasing the patients’ feeling of vulnerability and insecurity, the use of these devices and the unpredictability of its functioning can generate discomfort, implying care that may limit daily activities, requiring adjustments in routine and changes in lifestyle^[5]^.

Although PMs are currently highly reliable, environmental interferences can influence their functioning. The home environment rarely causes interference; however, some restrictions are recommended to the patient to ensure proper functioning, depending on the generation of the device. The new generation of PMs have a shielding that isolates the devices from most sources of interference^[6]^. Nevertheless, for older devices, it is recommended to avoid direct contact of the site where the PM generator is located with devices used in endodontics^[6]^, some running household appliances, metal detectors, and cell phones. This equipment must be kept at a distance of 10 to 15 cm from the PM^[7]^.

Despite recent advances in cardiac device technology, the stigma of having a device controlling the heart remains^[5]^. PM patients report fear of dependence on the device, which becomes responsible for stimulating their heartbeats, and distress at the thought that if the device fails, they may need emergency care to ensure life. Feelings of anguish and symptoms of anxiety and depression, reported by patients in a study conducted more than 20 years ago^[8]^, still accompany patients since the implantation of the device^[9]^.

With implantable cardioverter defibrillator (ICD) patients, this situation is no different^[9]^.

According to data from our national health system database (the Departamento de Informática do Sistema Único de Saúde, or DATASUS), in the year 2018, 1,736 ICDs were implanted in Brazil^[1]^. After ICD implantation, anxiety may be present and associated with the duration of the generator, fear of performing routine household activities, and also concerns about job loss, changes in sexual activity, loss of social, family, and professional statuses, perceived changes in self-image, and the feeling of early body deterioration^[10^,^11]^. Thus, despite the numerous proven clinical benefits, there is evidence that ICD can cause psychological adverse effects of varying degrees, which can impact the lifestyle and quality of life of individuals. The most commonly observed adverse effects are pain and discomfort, sleep disturbances, loss of libido and preoccupation with sexual activity, fatigue, changes in body perception, reduced physical and social activities, feelings of impaired independence, confrontation with one’s own death, expectations of ICD shocks, and concerns about device malfunction^[10]^.

The investigation of anxiety and depression symptoms in these patients, with PM or ICD and with or without a diagnosis of Chagas disease, is justified, considering that the presence of one of these devices requires attention in relation to care with the device. Chagas disease has among its consequences the probable development of chronic Chagas cardiomyopathy, which is perceived as a serious condition with the possibility of sudden death. Currently, Chagas disease still imposes on the affected individuals and, by extension, on their families^[11]^ a condition of vulnerability that often puts them in a situation of social and professional discrimination^[3]^. The experience of the disease in this case has exacerbated repercussions, since it is added to the treatment that, if on the one hand, brings benefits, on the other, brings concerns and exacerbates the stigma of the disease.

We found studies that evaluated the presence of anxiety and depression in patients with PM^[12]^; however, those comparing the intensity of these symptoms in the two groups, PM and ICD, are scarce^[9^,^13]^.

We did not find studies that investigated the relationship between the intensity of the symptoms of anxiety and depression according to a diagnosis of Chagas disease and the presence of one of these devices and the patient’s sex. Thus, the main objective of this study is to investigate the intensity of anxiety and depression symptoms in individuals with implantable cardiac PMs or ICDs and to explore the relationship of these symptoms with the presence or absence of a diagnosis of Chagas disease and the patient’s sex.

## METHODS

This is an observational, analytical, cross-sectional study. Data collection was carried out at the Arrhythmia Outpatient Clinic of a university hospital in the countryside of São Paulo. A consecutive and non-probabilistic sample was composed of patients of both sexes, older than 18 years of age, regardless of social class and race, with ICD or implantable cardiac PMs, in outpatient care on the day of data collection.

Patients who were not cognitively able to answer the questionnaires, who presented clinical decompensation of heart disease on the day of data collection, and patients with a cardiac resynchronizer or PM concomitant with ICD or with indication for concomitant use were excluded.

For the assessment of temporal and spatial awareness and orientation, six questions adapted from previous studies were used^[14]^, excluding those who got three or more of the following questions wrong or were unable to report: “What is today’s date?”, “What is your age?”, “What day of the week is today?”, “What is the name of the place we are now?”, “What is your full name?”, and “What is the name of the city in which you were born?”.

For sociodemographic and clinical characterization of the participants, an instrument developed by the researchers was used, which included the data described below: dates of birth and of interview (for later calculation of age, in years), sex, marital status, education, professional situation, and monthly family income. The clinical data collected were date of device implantation, diagnosis of Chagas disease, type of arrhythmia that indicated the device implantation, presence of associated diseases, and use of psychiatric drugs. Considering the specificity of each clinical condition that led to the indication of implantation of the different devices, the following data were also collected for patients with ICD: type of indication for implantation (primary or secondary prevention), family history of sudden death, and presence of shock. For patients with PM, the type of device (unicameral or bicameral) was investigated. The time to device implantation was calculated by subtracting the date of the interview from the date of implantation.

For the assessment of anxiety and depression symptoms, the Hospital Anxiety and Depression Scale (HADS) was used^[15]^, adapted to Portuguese^[16]^. The choice for these scale is justified because it has been widely used to assess symptoms of anxiety and depression in patients with heart disease^[17^,^18]^. HADS has 14 questions (seven for anxiety and seven for depression) that address somatic and psychological symptoms and a four-point response scale. The values of the answers vary from zero to three and the sum can vary from zero to 21 points, for each of the emotional disorders researched. Thus, in the present study, the answers will be evaluated with the total value of each subscale (HADS-anxiety and HADS-depression), and the higher the value, the higher the perception of anxiety and depression symptoms.

Data collection was performed on the pre-scheduled return day at the outpatient clinics of the hospital where the study was conducted, through individual interviews and consultation of participants’ medical records, in the period from April 2016 to July 2019, for patients with PM, and in the period from November 2018 to August 2019, for patients with ICD. The instruments were applied and data were recorded by two researchers in each group of patients.

The PM or ICD outpatient clinics where the data were collected see an average of 15 patients weekly, independently. After device implantation, regardless of being PM or ICD, the patient has the first return visit scheduled one week after implantation and, in the follow-up, at three, six, and nine months. If the patient has no intercurrence, the appointment becomes biannual, for control and follow-up of the device.

The results presented here are linked to two research projects prepared in accordance with the ethical precepts of National Health Council Resolution 466/12 and approved by the Research Ethics Committee of the Escola de Enfermagem de Ribeirão Preto, Universidade de São Paulo (CAEE: 49315415.5.0000.5393 and CAAE: 92179118.0.0000.5393). All patients were invited to participate in the research, and the free and informed consent term was read and signed.

Data was entered into the IBM Corp. Released 2013, IBM SPSS Statistics for Windows, version 22.0, Armonk, NY: IBM Corp. Descriptive analyses of simple frequency were performed for nominal or categorical variables, and analyses of central tendency (mean and median) and dispersion (standard deviation [SD]) were performed for numerical, discrete, and continuous variables. To compare the sociodemographic variables between the groups (patient’s sex, presence of partner, and professional situation) and clinical characterization (systemic arterial hypertension, atrial fibrillation, diabetes mellitus, dyslipidemia, and use of psychotropic drugs), we used the Chi-squared test. To compare the age, years of education, and monthly family income between the groups, we used the Student’s *t*-test. To investigate the possible relationship of anxiety and depression symptoms of patients with the type of device (PM or defibrillator, presence or absence of Chagas disease, and patient’s sex [male or female]), considering the scale score, we used the Student’s *t*-test. The level of significance was 0.05.

## RESULTS

A total of 168 individuals with PM and 76 with ICD were interviewed. The sociodemographic characterization of the 244 participants, according to the type of implanted device, is shown in Table 1. In the group of patients with PM, we observed a higher presence of women (*P*=0.034), with higher mean age (*P*<0.001), and low education (*P*=0.008) and family income (*P*=0.025), with these differences being statistically significant when compared to patients with ICD.

**Table 1:** Sociodemographic characterization of the 244 participants according to the type of device (implantable cardiac pacemaker and implantable cardioverter defibrillator). Ribeirão Preto, 2019.

Variable	Pacemaker (n=168)	Defibrillator (n=76)	*P*-value
Sex, n (%)			0.034*
Female	91 (54.2)	30 (39.5)	
Male	77 (45.8)	46 (60.5)	
Presence of a partner, n (%)			0.306*
Yes	98 (58.3)	39 (51.3)	
No	70 (41.7)	37 (48.7)	
Professional status, n (%)			0.844*
Inactive	133 (79.2)	61 (80.3)	
Active	35 (20.8)	15 (19.7)	
Age, mean (SD)	64.8 (15.3)	53.4 (13.9)	< 0.001^#^
Complete education in years^¶^, mean (SD)	5.5 (4.0)	7.0 (4.4)	0.008^#^
Monthly family income in Reais^a^, mean (SD)	1.865.9 (1.357.4)	2.283.0 (1.013.5)	0.025^#^

* Chi-squared test

# Student’s *t*-test

¶ 157 pacemaker patients and 74 cardioverter defibrillator patients

a 167 pacemaker patients and 66 cardioverter defibrillator patients SD=standard deviation

The clinical characterization of the participants by device group is shown in Table 2. Of the 168 individuals with PM, 85 (50.6%) had been diagnosed with Chagas disease, and of the 76 with ICD, 19 (25%) had Chagas disease.

**Table 2:** Clinical characterization of the 244 participants according to the type of device (implantable cardiac pacemaker and implantable cardioverter defibrillator). Ribeirão Preto, 2019.

Variable	Pacemaker (n=168)	Defibrillator (n=76)	*P*-value
	n (%)	n (%)	
Systemic arterial hypertension (yes)	77 (45.8)	33 (43.4)	0.726*
Atrial fibrillation (yes)	31 (18.4)	-	< 0.001*
Diabetes mellitus (yes)	30 (17.8)	12 (15.8)	0.724*
Dyslipidemia (yes)	19 (11.3)	20 (26.3)	0.003*
Use of psychotropic drugs (yes)	38 (22.6)	6 (7.9)	0.005*
*Chi-squared test			

In the group of patients with PM, we observed a higher presence of atrial fibrillation (*P*<0.001) and higher use of psychotropic drugs (*P*<0.001), with these differences being statistically significant when compared to patients with ICD. On the other hand, in the group of patients with ICD, we observed a higher presence of dyslipidemia (*P*=0.003), with this difference being statistically significant when compared to patients with PM.

The main implanted PM was the bicameral type (153; 91.9%), and the mean time of implantation was 117.5 months (SD=103.6; median=86.5), ranging from one to 491 months. In the medical records of the 168 patients with this device, we identified the following indications: complete atrioventricular block (AVB) (59; 35.1%), sinus node disease (11; 6.6%), second degree AVB (10; 5.9%), bundle branch block (3; 1.8%), and first degree AVB (1; 0.7%). This information was absent in the records of 50% of the patients interviewed (n=84).

Among the 76 patients with ICD, 56 (73.7%) had the device implanted as a primary prevention measure, and the others as secondary prevention after cardiac arrest. And among them, we found as an indication for the use of the device the presence of ventricular tachycardia (30; 39.5%) and ventricular fibrillation (9; 11.8%). There was no such information in 37 medical records (48.7%). Thirty-nine (51.3%) patients had a past family history of sudden death, and eight (10.5%) reported experiencing shock. The mean time of ICD implantation was 54.4 months (SD=46.2; median=48.0), ranging from less than one month to 196 months, and the difference was statistically significant (*P*<0.000) when compared with the mean time of PM implantation.

The comparison of anxiety and depression symptom measures according to the sex of the participants, type of device (with PM or ICD), and the diagnosis of Chagas disease are described in Table 3.

**Table 3:** Comparison of the means of the subscales of the Hospital Anxiety and Depression Scale (HADS) instrument according to the sex of the participants, type of device, diagnosis of Chagas disease, and probability values (*P*) associated with Student’s *t*-test. Ribeirão Preto, 2019.

Variable	HADS -anxiety Mean (SD)	HADS -depression Mean (SD)
Sex		
Female (n=121)	7.0 (4.3)	6.3 (4.4)
Male (n=123)	5.3 (4.0)	4.4 (3.5)
*P*-value	0.002	< 0.001
Type of device		
Pacemaker (n=168)	6.1 (4.1)	5.7 (4.0)
Defibrillator (n=76)	6.4 (4.5)	4.6 (4.3)
*P*-value	0.594	0.071
Chagas disease		
Yes (n=104)	6.3 (4.1)	5.6 (3.7)
No (n=140)	6.1 (4.3)	5.2 (4.3)
*P*-value	0.649	0.354
SD=standard deviation		

We found that women had higher means than men for both anxiety (*P*=0.002) and depression (*P*<0.001). We found no statistically significant differences in mean scores for anxiety and depression when comparing participants according to the type of device and diagnosis of Chagas disease.

## DISCUSSION

In this study, we did not find statistically significant differences in the measurements of anxiety and depression symptoms when comparing the participants according to the type of cardiac device implanted for the treatment of arrhythmia and the presence of Chagas cardiomyopathy. Compared to the ICD group, the PM group presented with a greater number of women, with higher mean age, and low education and family income, with the differences being statistically significant.

The mean scores for anxiety and depression found in our study according to the type of device implanted are similar to those found by other authors^[13]^, which also used HADS to assess these symptoms, although in our study, the mean time since the installation of PM and ICD was longer than in that previous study^[13]^.

In a study of 296 Iranians, levels of anxiety and depression were compared among 98 patients with PM, 100 with ICD, and 98 individuals in the general population using the Beck Anxiety and Depression Inventories. There were no significant differences in anxiety levels between the PM and ICD groups; however, participants in both groups had significantly higher levels of anxiety and depression than the general population. For these two groups, anxiety was rated as moderate. In the comparison between the groups, with PM and ICD, individuals with PM had significantly higher levels of depression^[9]^.

The women participating in our study had significantly more anxiety and depression symptoms than the men in both groups (with PM or ICD). Results from a study of 250 individuals with PM also showed women with higher levels of anxiety symptoms than men^[19]^. According to a study of patients with grades I and II of heart failure, female sex is associated with high levels of anxiety and depression^[20]^. Other authors have found a higher propensity for anxiety among women with ICDs compared to men, regardless of whether they are shocked by the presence of the device^[21]^.

In an investigation on the relationship between the prevalence of ICD concerns, the experience of shock, and the relationship of these manifestations with the presence of anxiety and depression symptoms, other authors found that ICD concerns were the only independent determinant of the presence of anxiety and depression symptoms. In this study, anxiety and depression were assessed by HADS, using as cutoff score values ≥ 8^[22]^. Patients who experienced shocks, appropriate or not, showed high levels of concern about ICD^[22]^ and anxiety symptoms^[23]^. In the latter study, the authors used the Florida Shock Anxiety Scale to assess anxiety and compare patients regarding the experience of shock. They identified that the presence of multiple shocks in patients with ICD was associated with higher levels of anxiety at nine months of follow-up^[23]^. In our study, only 10.5% of ICD patients reported the perception of shock.

For most individuals participating in our study, in both groups (PM and ICD), the mean intensity of anxiety and depression symptoms was < 9, the threshold value adopted to designate the presence of anxiety or depression in methodological investigations of the psychometric validity of HADS^[15]^. In a study carried out in Geneva, Switzerland, with 137 Latin American patients living in that country diagnosed with Chagas disease, a higher percentage of patients presented with anxiety (58.4%) and a lower percentage with depression (28.5%). However, the cutoff point adopted by the authors was < 8 for the measures of anxiety and depression symptoms^[24]^.

Among the participants, half of the patients with PM and a quarter of the patients with ICD had a diagnosis of Chagas disease. This result reflects the prevalence of the disease in patients seen in this hospital, many of them from rural areas of small cities in the states of São Paulo and Minas Gerais. This disease, considering those associated with heart problems, occupies an important position in some regions of Brazil, leading, in many cases, to the indication of PM or ICD implantation^[4]^. It is characterized as a serious disease that affects the gastrointestinal system and, frequently, the heart of infected individuals, with consequences for the conduction of the cardiac stimulus and for the myocardium. It is characterized as a condition that is associated with a feeling of vulnerability due to limitations and the threat of death, mainly because the diagnosis is often made late, when symptoms appear. Patients report insecurity and depression when facing the situation^[8]^, which becomes even more ominous when informed of the need to install a device that will “control the heart”.

A study of patients with PM, with or without a diagnosis of Chagas disease, showed that participants with the disease had a worse prognosis, with a lower left ventricular ejection fraction, and a higher incidence of arrhythmias in 24 hours, when compared to those without the disease^[25]^. Although this aspect was not investigated in this study, the presence of a diagnosis of Chagas disease and devices (PM or ICD) could be perceived by patients as an indication of greater severity that could lead to an average of anxiety and depression symptoms compared to those who had the implant indicated for other etiologies. In a study carried out with Brazilian patients with heart failure, it was found that high levels of anxiety and depression symptoms are associated with the diagnosis of Chagas disease^[20]^. According to the authors of this study, Chagas disease increases levels of anxiety and depression by 44% and 41%, respectively^[19]^.

The results of our study may have been influenced by the sample size and by the fact that the groups studied, PM and ICD, presented significantly differences in relation to sociodemographic characteristics and some clinical situations, which may have an influence on the severity of the individual’s condition. However, our results also showed that the participants with PM and with Chagas disease and in use of psychotropic drugs are higher in number, which could also indicate a worse mental state, when compared to those with this device and without this diagnosis.

### Limitations

This study has limitations related to the size and sociodemographic characteristics of the sample studied, arising from the fact that the participants were recruited from a service that serves patients of the Brazilian unified health system, which serves mainly individuals belonging to the lower income brackets. The information collected from the patients’ electronic medical records was limited by the sometimes incomplete records. Despite the limitations that hinder the generalizations of its results, this study presents contributions to the nursing area, reinforcing the need for interventions to minimize the symptoms of anxiety and depression in patients with PM and ICD, with special attention to women. Knowing the intensity of anxiety and depression symptoms in patients with Chagas disease or not and with PM or ICD is important for nurses to plan care for these groups and their families, considering the specificities of each one.

## CONCLUSION

The results of our study showed that participants with PM and ICD did not present significant differences in relation to anxiety and depression symptoms. The same occurred in relation to the diagnosis of Chagas disease. We found higher mean scores for anxiety and depression symptoms among women.

Further studies should explore this, considering the aspects involving the relationship between these symptoms in women and self-care in the presence of these devices (PM and ICD).

**Table:** 

Authors’Roles & Responsibilities	
CAM	Substantial contributions to the conception or design of the work; or the acquisition, analysis, or interpretation of data for the work; drafting the work or revising it critically for important intellectual content; agreement to be accountable for all aspects of the work in ensuring that questions related to the accuracy or integrity of any part of the work are appropriately investigated and resolved; final approval of the version to be published
EMOG	Substantial contributions to the conception or design of the work; or the acquisition, analysis, or interpretation of data for the work; drafting the work or revising it critically for important intellectual content; agreement to be accountable for all aspects of the work in ensuring that questions related to the accuracy or integrity of any part of the work are appropriately investigated and resolved; final approval of the version to be published
IBI	Substantial contributions to the conception or design of the work; or the acquisition, analysis, or interpretation of data for the work; drafting the work or revising it critically for important intellectual content; agreement to be accountable for all aspects of the work in ensuring that questions related to the accuracy or integrity of any part of the work are appropriately investigated and resolved; final approval of the version to be published
PAF	Substantial contributions to the conception or design of the work; or the acquisition, analysis, or interpretation of data for the work; drafting the work or revising it critically for important intellectual content; agreement to be accountable for all aspects of the work in ensuring that questions related to the accuracy or integrity of any part of the work are appropriately investigated and resolved; final approval of the version to be published
SROM	Substantial contributions to the conception or design of the work; or the acquisition, analysis, or interpretation of data for the work; drafting the work or revising it critically for important intellectual content; agreement to be accountable for all aspects of the work in ensuring that questions related to the accuracy or integrity of any part of the work are appropriately investigated and resolved; final approval of the version to be published
LAR	Substantial contributions to the conception or design of the work; or the acquisition, analysis, or interpretation of data for the work; drafting the work or revising it critically for important intellectual content; agreement to be accountable for all aspects of the work in ensuring that questions related to the accuracy or integrity of any part of the work are appropriately investigated and resolved; final approval of the version to be published
RASD	Substantial contributions to the conception or design of the work; or the acquisition, analysis, or interpretation of data for the work; drafting the work or revising it critically for important intellectual content; agreement to be accountable for all aspects of the work in ensuring that questions related to the accuracy or integrity of any part of the work are appropriately investigated and resolved; final approval of the version to be published
